# The development of dynamic perceptual simulations during sentence comprehension

**DOI:** 10.1007/s10339-020-00959-7

**Published:** 2020-02-21

**Authors:** Juliane E. K. Hauf, Gerhild Nieding, Benedikt T. Seger

**Affiliations:** Department of Psychology (Developmental Psychology), University of Würzburg, Röntgenring 10, 97070 Würzburg, Germany

**Keywords:** Embodied cognition, Sentence comprehension, Dynamic perceptual simulation, Children, Adults

## Abstract

Based on an embodied account of language comprehension, this study investigated the dynamic characteristics of children and adults’ perceptual simulations during sentence comprehension, using a novel paradigm to assess the perceptual simulation of objects moving up and down a vertical axis. The participants comprised adults (*N* = 40) and 6-, 8-, and 10-year-old children (*N* = 116). After listening in experimental trials to sentences implying that objects moved upward or downward, the participants were shown pictures and had to decide as quickly as possible whether the objects depicted had been mentioned in the sentences. The target pictures moved either up or down and then stopped in the middle of the screen. All age groups’ reaction times were found to be shorter when the objects moved in the directions that the sentences implied. Age exerted no developmental effect on reaction times. The findings suggest that dynamic perceptual simulations are fundamental to language comprehension in text recipients aged 6 and older.

**Electronic supplementary material:**

The online version of this article (10.1007/s10339-020-00959-7) contains supplementary material, which is available to authorized users.

## Introduction

Current models of text comprehension posit that recipients build a mental representation of not only the text itself but that they also elaborate on ideas beyond the explicit text. The result is a coherent, elaborated representation of the described state of affairs, referred to as a *situation model* (Zwaan and Radvansky 1998). The nature of such models has been debated. One line of research suggests that the information is stored as propositions with amodal characteristics. Here, propositions are the smallest units to which one can assign a truth value and are organized in a predicate–argument structure (e.g., Christmann 2004, 2006; Fletcher 1994; Hemforth and Konieczny 2008; Kintsch 1974; van Dijk and Kintsch 1983), in which the semantic content acquires its meaning from the connections between these propositions (Kintsch 1988; Kintsch and van Dijk 1978). Another line of research suggests that mental representations have an analog relation to external or real-world referents that have modal characteristics (Barsalou 1999; Glenberg 1997; MacWhinney 1999; Zwaan 1999). The basic assumption of theories informed by this embodied account of language comprehension is that the mental representations generated during language comprehension are grounded in perception and action. In keeping with this view, language-based representations are similar to the representations generated during real-life experiences because both depend on the same (modality-specific) systems. This echoes the idea of a fundamental mechanism in which simulations during language comprehension are reenactments of activities in sensory motor brain areas that are extracted and stored in memory when a person interacts with his or her environment (Barsalou 1999).

A host of studies—both behavioral and neuroscientific—supports the embodied view of language comprehension in adults. For example, Stanfield and Zwaan (2001) demonstrated that language comprehension incorporates earlier perceptual experiences. They employed a perceptual mismatch paradigm to show that adults mentally simulate the orientation of objects during sentence processing, thereby implying that people generate static visual representations. In the study conducted by Stanfield and Zwaan (2001), the participants read sentences describing objects at certain locations in a way that implicitly specified the objects’ vertical or horizontal orientations. After reading a sentence, each participant viewed a picture that showed the depicted object in a vertical or horizontal orientation and then pressed a key to indicate whether it had been mentioned in the sentence (i.e., *picture verification task*). In accordance with a modal view of language processing, the results showed that the reaction times were shorter for matching (e.g., implied vertical orientation of the object in the sentence and vertical orientation in the picture) than for mismatching trials. Other studies using this paradigm have shown that adults also simulate the shapes of objects (e.g., Zwaan et al. 2002), as well as their colors (Therriault et al. 2009). Perceptual simulations also have been found in the auditory modality, indicating that they extend beyond the visual (Brunyé, et al. 2010). Neuroscientific studies support this claim: During the processing of words and sentences, activated cortical areas correspond to the sensory modality that is verbally described (e.g., Bastiaansen et al. 2008; González et al. 2006).

In addition to *perceptual* simulations, behavioral studies have shown that adults reenact *motor* traces during language comprehension (Glenberg and Kaschak 2002; Glenberg et al. 2008; Zwaan and Taylor 2006). This is supported by neuroscientific studies that have found the activation of cortical areas associated with the foot, hand, and face when individuals processed these body parts’ respective verbs (Buccino et al. 2005; Pulvermüller 2005; Tettamanti et al. 2005). Furthermore, there is evidence that the understanding of emotion-related descriptions is also *grounded* (Barrett 2006; Glenberg et al. 2005; Havas et al. 2007). The fact that even nonperceptual referents are simulated during language comprehension in adults suggests that simulations are the building blocks of situation models.

Unlike adult studies, research on children’s mental representations during language comprehension remains underanalyzed, with one unresolved question concerning whether children’s situation models also are grounded in perception and action. Preliminary indications suggest that this may be so. One early study replicating the method and results for adults showed that 8–13-year-old children also mentally simulate the shapes and orientations of objects during sentence comprehension (Engelen et al. 2011). Predictive inferences also seem to be grounded in simulation. For instance, when a 6-year-old child sees someone who is about to blow up a balloon, he or she simulates the balloon’s inflated state (Author 2009).

Wellsby and Pexman (2014) emphasized the role of prior experience in simulating objects. They investigated word naming latency and accuracy by using the *body*–*object interaction* (BOI) concept. If a word has a high BOI, this means that one easily can interact physically with its referents. For example, a belt’s BOI is high but not that of a roof. In Wellsby and Pexman’s study, high BOI words were associated with faster naming latency and accuracy in children aged 8 years and older, as well as young adults, but not in younger children aged 6–7 years.

Additional studies have shown that simulations not only play a role in online text comprehension, but also enhance children’s memory of story content when they are given sensory motor access to the described situation, for example handling relevant toys (Glenberg et al. 2007; Marley et al. 2010) or plastic figures on a storyboard (Rubman and Salatas Waters 2000).

## Dynamic character of situation models

Other studies of the dynamic character of situation models investigating objects’ visual motions or a person’s motor actions also provide evidence of an embodied account of language processing. Within the traditional view of imagery, the dynamic character of mental representations has been postulated and demonstrated. Freyd and Finke (1984) coined the term *representational momentum* to refer to how people mentally perceive the implied motions of objects in photographs along their trajectories. These and other imagery studies argue for the existence of mental simulations in the experimental tradition of Shepard and colleagues, who explored the mental rotation of single objects in three-dimensional space (e.g., Shepard and Metzler 1971). In studies of this kind, participants had to decide whether two drawn objects were the same when these objects were rotated to specific angles. The results showed that response latencies increased as a function of the difference in angles between the two objects. Such an effect could not be explained readily by the propositional models suggested, for example, by Pylyshyn (1981). More recently, Iachini (2011) pointed out that mental imagery can be seen as an instance of perceptual simulation. In her literature review, she also invoked several arguments that favor analog over propositional views. One prominent argument stems from neuroimaging research, namely, that most of the same neural processes underlying perception or motor control are also used in visual or motor mental imagery (see also Borst 2013).

Even if the simulation process, which is underlying mental imagery, is rather voluntary because it requires conscious control (Iachini 2011), this research domain has formed the background for the exploration of dynamic perceptual simulation (Zwaan et al. 2004). In Zwaan et al. (2004) study, the participants listened to sentences that described a ball flying toward or away from their perspectives (e.g., “Scott smacked the ping pong ball toward you”). Thereafter, the participants were shown two pictures of a ball, one after the other, in which the second ball was either bigger or smaller than the first and hence seemed to move toward or away from the participants. The task was to state whether the two pictures referred to the same object. This task was performed more quickly when the directions of motion implied by the sentences matched the directions of motion in the pictures, supporting the argument for dynamic mental representations during language comprehension.

Evidence also exists that people mentally simulate incidents on the horizontal or vertical axes based on the use of descriptive verbs (Richardson et al. 2003). They simulated not only the axis, but also the object’s specific location and direction of motion (up or down) on this axis (Bergen et al. 2007). Other studies have revealed dynamic simulations of motion during language comprehension, further supporting the assumption that verbal descriptions of motion are grounded in perception and action (Kaschak et al. 2005; Meteyard et al. 2007; 2008). In a motion detection task in Meteyard et al. (2007) study, while listening to verbs describing an upward or downward motion, participants had to detect the motions of visual stimuli near the threshold level that moved up or down the vertical axis. The results showed that the participants had difficulty detecting these motions (i.e., slower reaction times) when the verbs they heard were incongruent with the motion signals (e.g., when the verb *fall* was combined with visual stimuli moving upward). Eye tracking has also been used to provide evidence of the simulation of dynamic motion information. Speed and Vigliocco (2014), for example, discovered that eye movements are affected by the implied speed of a verb. Their adult participants listened to sentences that contained either a fast (e.g., dash) or slow (e.g., amble) verb while they were looking at graphics that contained the agent and destination of the sentence, as well as a path between them. As a result, slow verbs were associated with longer dwelling times on the agent or the destination compared with fast verbs.

This brief review of more recent experimental research confirms that adults’ situation models are multifaceted and quite elaborate. Current research also endorses modal theories of adults’ language comprehension as grounded in perceptual motor simulations, and evidence suggests that these mental representations are dynamic in character and incorporate changes over time. However, to date, no study has investigated dynamic aspects in children’s situation models by exploring mental representations related to objects’ motion. In the following section, we discuss the current state of research, including motion aspects in children’s situation models. Given the lack of research in this area, we also consider studies on protagonists’ motions.

## Motion aspects in children’s situation models

To date, few studies have investigated the role of dynamic perceptual simulation during language comprehension. In Fecica and O’Neill’s (2010) study, children represented how long a protagonist’s movement lasted, depending on whether the protagonist was walking or driving. The 4- and 5-year-old participants heard sentences and proceeded through the story by clicking a mouse button (as an analog for self-paced reading latencies). Sentences that contained the word “walking” attracted longer latencies than those that contained “driving.” These results suggest that the children simulated the protagonist’s implicit motion speed based on the description in the text.

Another line of research (Author 1999; 2006) investigated the updating of character movements in the spatial situation models of 6–7-year-old children based on an adaptation of Bower and Morrow’s (1990) classical paradigm. After learning the layout of a building, including the arrangement of rooms and the objects they contained, the children were told a story in which a protagonist moves from a *source room* through a *path room* to a *goal room*. The path room, which the protagonist had to pass through, was not mentioned in the story but could be inferred from the previously learned layout. After hearing the story, the participants were shown two pictures and had to decide whether the objects depicted belonged in the same room. Their reaction times showed that the path room was accessed the most readily. A study involving 9–16-year-old participants corroborates these findings: The proportion of correct answers was significantly higher for objects in the path room compared with those in the source or goal rooms, although no difference in reaction times was reported (Barnes et al. 2014). Among children, the higher cognitive availability of the path room compared with the goal room contrasts with the results for adults, for whom the goal room cognitively was the most available (Bower and Morrow 1990). We assume that because of their lower processing speed in general (e.g., Kail and Salthouse 1994), children also process information about motion more slowly and thus “stay” longer in the path room than adults. In addition to Fecica and O’Neill’s (2010) findings, these studies confirm that children also simulate the protagonists’ implicit motions when constructing complex spatial situation models.

That children as young as those in the Fecica and O’Neill (2010) study engage in a process of mental simulation during text comprehension indicates that protagonists play a crucial role in generating situation models among children of early childhood age. This is supported by other studies of situation models that include protagonists. For example, in Nyhout and O’Neill’s (2013) study, 7-year-old children heard narratives about a particular neighborhood layout. Half the children became familiar with the spaces after hearing a narrative in which a goal-driven protagonist walked through the neighborhood. The other children, by contrast, achieved this after hearing only a description of the neighborhood layout. The stories’ critical content was similar. Thereafter, the children had to recreate the neighborhood using miniature models. The results showed that the children constructed better spatial situation models under the narrative condition that included the protagonist.

Research on the simulation of motion aspects in children’s language comprehension remains at an early stage, and studies have focused on moving people rather than objects. By comparison, studies involving adults are more advanced, and several have revealed dynamic perceptual simulations in adults (e.g., Zwaan et al. 2004). Although the available evidence suggests that children perceptually simulate static object features during text comprehension, their dynamic perceptual simulations of objects’ motions have not yet been investigated. In addition, little is known about the developmental time course of embodied cognition. For instance, at what age does one perceptually simulate motion directions during language comprehension? Does the role of perceptual and motor information in language comprehension change during children’s development?

To explore these questions, in the present study, we asked both children and adults to complete the same task—a methodological strengthening that should facilitate the exploration of the developmental course of embodied cognition. We deemed a picture verification task suitable because this approach already has been validated for children (Engelen et al. 2011) and adults (e.g., Zwaan and Pecher 2012) but has not yet been used to assess the simulation of vertical object movements in either age group. Here, the questions related to whether an illustrated object had been mentioned in a previously heard German sentence. The said object moved either down or up and stopped in the middle of the screen; its direction of motion either matched or mismatched the direction that the preceding sentence had implied. Perceptual simulation should be indicated by shorter reaction times in the matching trials. As mentioned above, we were also interested in the development of dynamic perceptual simulation. Therefore, we included adults and children who were aged between 6 and 10. We assumed that the children and adults could mentally represent the objects’ motion direction.

## Method

### Participants

Of the 156 children and adults who participated in the study, 39 were 6-year-old children (*M*_age_ = 6;0, SD_age_ = 0;3, female = 20, male = 19), 38 were 8-year-old children (*M*_age_ = 8;1, SD_age_ = 0;4, female = 20, male = 18), 39 were 10-year-old children (*M*_age_ = 10;0, SD_age_ = 0;4, female = 19, male = 20), and 40 were adults (*M*_age_ = 22;1, SD_age_ = 4;4, female = 32, male = 8). We recruited the children from kindergartens and schools, and the adults were university psychology students. All participants provided informed consent. For the participating children, informed consent forms signed by their parents were required and obtained.

## Materials

In a pilot study, we tested whether children in our studied age range would be familiar with the objects that we planned to use as stimulus material. To this end, 18 children (six 6-year-old children, six 8-year-old children, and six 10-year-old children, each group comprising three girls and three boys) were asked to name the objects illustrated in the pictures. In some cases, the objects that proved to be unknown to most of the children were excluded from the main study. In other cases, we changed the labels when the children used other labels (e.g., the German label *Teddybär* for *Kuschelbär* [in English: *teddy bear*]).

Based on this pilot study, we selected 36 objects for 36 experimental sentences. Eighteen of these sentences implied a vertical upward motion (e.g., “Die Rakete fliegt hoch ins Weltall” [“The rocket takes off into space”]), and 18 implied a vertical downward motion (e.g., “Die Schatztruhe sinkt herab auf den Meeresboden” [“The treasure chest sinks to the seabed”]). The sentences were presented in either a matching or mismatching mode (variation in compatibility). *Matching* implied that the object moved in the same direction in the sentence as in the picture, whereas *mismatching* meant that they moved in the opposite directions, respectively.

All the experimental sentences required an affirmative response. We also constructed 56 filler sentences that were analogous to the experimental sentences. Ten of these filler sentences required an affirmative response and targeted the last object in the sentence to prevent the participants from attending only to the object noun and not to the entire sentence. For example, the sentence “Maria sees a dog” was accompanied by the picture of a dog. In the remaining 46 filler sentences, the target object was not part of the sentence; therefore, a “no” response was required, yielding an equal number of “yes” and “no” answers. Additionally, the filler sentences were used to conceal the experimental sentences’ purpose because the former included types of motion (e.g., jump, roll, and swim), states (e.g., is, stand, lie), and other circumstances (e.g., shake, push, smell). The original German sentences and their English translations are found in Appendix.

The sentences were spoken by a native German-speaking woman. Using Audacity^®^ software (Audacity Team, version 2.0.2), we edited the sentences so that the recording terminated at the end of the final word. The average length of each sentence was 2.83 s, with an average word count of 6.5 words per sentence. The WAX^®^ program (Debugmode, version 2.0) was used to animate the objects’ vertical motion. The display background was white, and the pictures were in color.

### Procedure

**Fig. 1:**
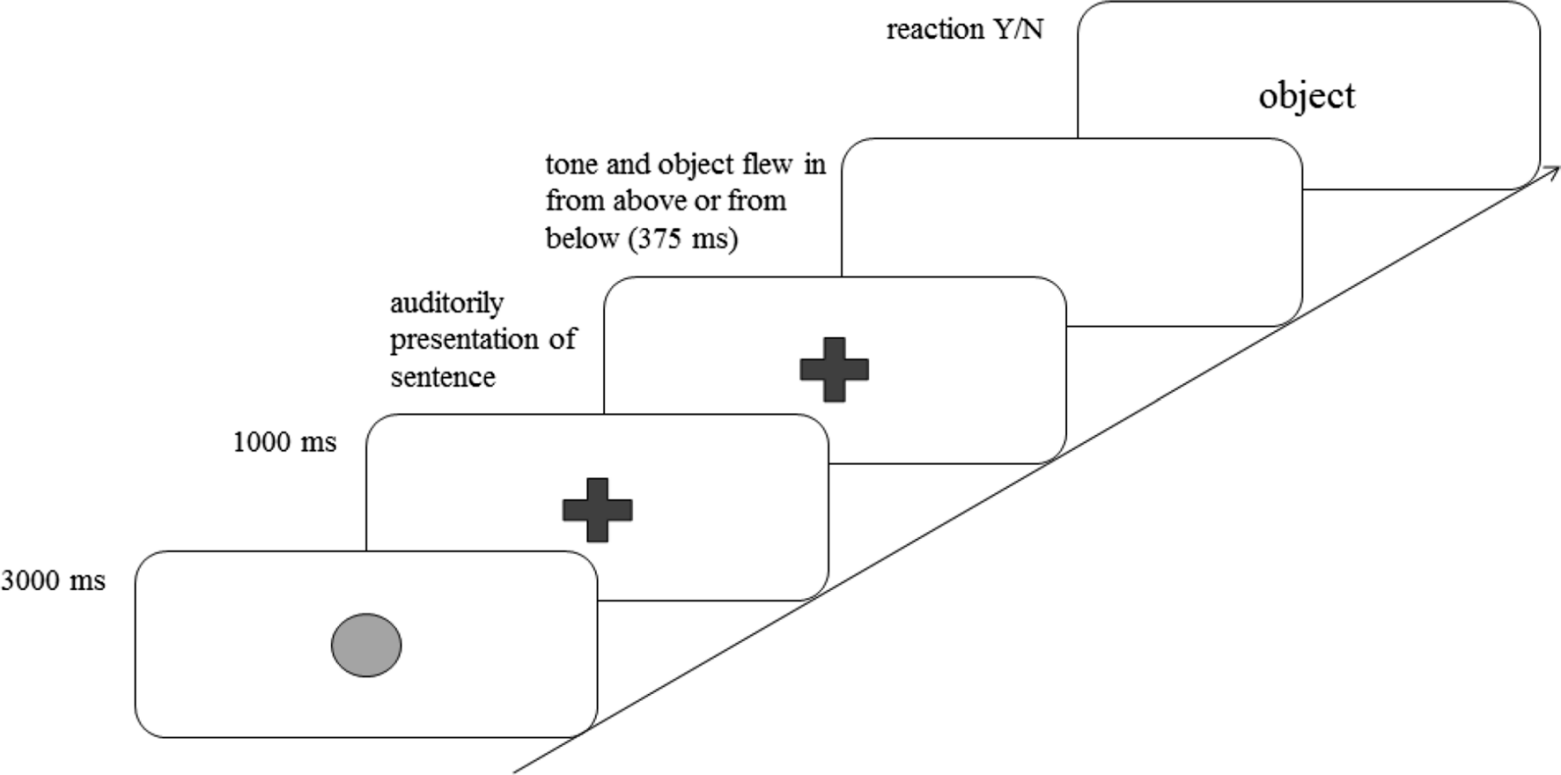
Event sequence of a sample trial. In the experiment, the target objects were colored, the dot was blue, and the cross was green

Each trial, including the filler trials, began with a blue dot, which was shown for 3000 ms (ms); then, a green fixation cross appeared in the middle of the screen for 1000 ms, cueing the beginning of the next trial (see Fig. 1). The fixation cross remained on the screen while the sentence was presented. As soon as the sentence ended, a short tone sounded, and the fixation cross disappeared; this signaled that the picture would now enter the screen. The object moved either downward or upward and stopped in the middle of the screen where the fixation cross had been. This took 375 ms, and the participants had to decide as quickly as possible whether this object was part of the sentence, which they indicated by pressing the *yes* or *no* button on an external keyboard. The *yes* button depicted a happy emoticon, while the *no* button depicted an unhappy emoticon. We used emoticons to ensure that preliterate children would be able to comply with the instructions. Emoticons have also been employed in other language comprehension studies with children (e.g., Author 2015; Cummings and Čeponiene 2010). The Presentation^®^ program (Neurobehavioral Systems, version 16.3) was used to record the participants’ reaction times and their answers’ correctness or lack thereof.

We constructed two different test conditions (A and B) for the experimental sentences: The 18 experimental sentences that matched in test condition A did not match in test condition B, and the remaining 18 experimental sentences that did not match in test condition A matched in test condition B. Hence, each participant responded to 18 matching and 18 mismatching sentences. The filler sentences were the same in each test condition. In addition, two versions (1 and 2) of test conditions A and B were used, in which the orders of the sentences differed. Finally, we varied the position of the *yes* and *no* buttons (right- or left-hand side) equally across conditions and versions. The participants were randomly assigned to one of these eight test variants.

Each participant was tested during a single session. Before the experiment commenced, the participants underwent eight practice trials. The experimental task consisted of 92 trials in total (36 experimental and 56 filler trials) that were divided into six blocks of 15 or 16 trials each. Between these blocks, the participants were given a short break. The whole procedure lasted approximately 30 min.

## Results

### Preliminary analyses

In the first analysis stage, two 6-year-old children were excluded because they provided a large number of incorrect responses (more than 25%) in total. Overall, the mean number of errors for both the filler and experimental sentences was very low (6-year-old children 5.49% [SD = 3.96%]; 8-year-old children 2.86% [SD = 2.49%]; 10-year-old children 2.51% [SD = 2.63%]; adults 2.72% [SD = 3.68%]).

Because the filler sentences were excluded, the analysis only included the experimental sentences. All extreme reaction times (i.e., those exceeding 5000 ms) and all incorrect responses were discarded. We then determined the mean reaction times for each age group and excluded those exceeding three standard deviations higher or lower than the mean for that age group (Rey 2012). This double procedure was used because some reaction times exceeded a participant’s range of all other reaction times by far, so we were concerned that they would seriously bias that participant’s mean reaction time. We also doubted whether perceptual simulation would play an essential role for more than five seconds after picture presentation. If more than 25% of a participant’s experimental trials were incorrect or discarded for another reason described above, this participant was excluded from further analysis. This concerned 9 participants in the second analysis stage (6-year-old children *n* = 3; 8-year-old children *n* = 2; 10-year-old children *n* = 2; adults *n* = 2). Hence, 11 participants were excluded in total. For the remaining participants, 93% of the data was entered for the main analysis (6-year-old children 89.62% [SD = 7.15%]; 8-year-old children 94.98% [SD = 5.59%]; 10-year-old children 95.05% [SD = 5.40%]; adults 90.57% [SD = 5.22%]). A repeated measure analysis of variance (ANOVA) with compatibility (matching vs. mismatching) and movement direction (up vs. down) as predictors did not indicate an unbalanced distribution of missing reaction times across the conditions (*p* > . 05 for both main effects and the interaction).

### Analyses of reaction times

Prior to our main analysis, we investigated whether the sentences themselves had an influence on the reaction times. For this purpose, we determined first whether sentence length (word count) correlated with the participants’ reaction times. This was not the case for all 92 sentences (*r* = .129, *p* = .219) or for the 46 sentences that required a positive answer only (*r* = .218, *p* = .145). Hence, sentence length was not included in further analyses. Then, a mixed ANOVA with compatibility (matching vs. mismatching) as within-participant factor and condition (A vs. B) as between-participant factor was run. The results indicate no main effect of condition (*F* < 1) and, most importantly, no interaction (*F* < 1). Therefore, we did not include conditions into the main analysis.

We conducted a 2*2*2*4 mixed ANOVA with compatibility (matching vs. mismatching) and movement direction (up vs. down) as within-subject factors and gender and age group (6-year-old children, 8-year-old children, 10-year-old children, and adults) as between-subject factors. A significant main compatibility effect existed (*F*[1, 137] = 9.880, *p* = .002, partial η^2^ = .067), revealing shorter reaction times in the matching trials (*M* = 1156 ms, SD = 447 ms) than in the mismatching trials (*M* = 1182 ms, SD = 445 ms). Unexpectedly, the main effect of movement direction was significant (*F*[1, 137] = 7.097, *p* = .009, partial η^2^ = .049), with faster reaction times for objects that moved up (*M* = 1156 ms, SD = 436 ms) rather than down (*M* = 1181 ms, SD = 457 ms). The within-participant factors did not interact (*F *< 1).

**Fig. 2:**
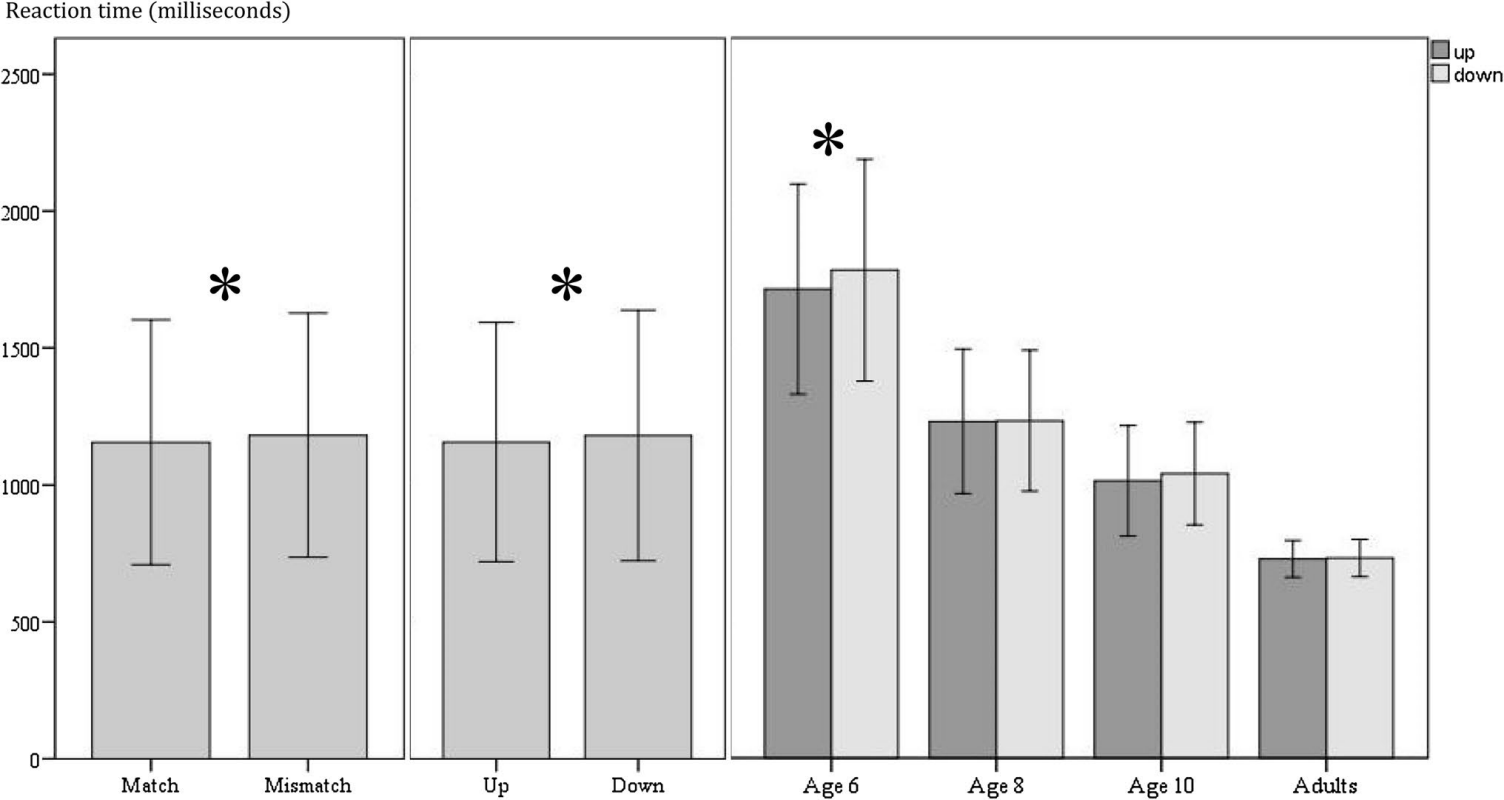
Mean reaction times in milliseconds according to the significant main effect of compatibility, main effect of direction, and direction*age interaction. Asterisks refer to significant mean differences (*p* < .05). Error bars represent standard deviations

The main effect of age group was significant (*F*[3, 137] = 96.897, *p* < .001, η^2^ = .680). Bonferroni-adjusted post hoc tests showed significant differences between all age groups (*p*s < .01). Reaction times decreased with age, which supposedly reflects the development of processing speed (Kail and Salthouse 1994). The interaction of age with compatibility was not significant (*F *< 1), so age does not seem to be related to the development of perceptual simulation. However, age interacted with movement direction (*F*[3, 137] = 3.256, *p* = .024, partial η^2^ = .067). Bonferroni-adjusted post hoc comparisons showed that only the youngest group exhibited significantly faster reaction times when the object in the sentences moved up (*M* = 1713 ms, SD = 385 ms) rather than down (*M* = 1783 ms, SD = 406 ms, *p* < .001). The three-way interaction of age, compatibility, and movement direction was not significant (*F *< 1). The significant main effects of compatibility, movement direction, and the interaction of movement direction with age are depicted in Fig. 2.

There was also an unpredicted main effect of gender (*F*[1, 137] = 10.183, *p* = .002), indicating that the reaction times of the male participants were faster than those of female participants. Gender did not interact with compatibility (*F*[1, 137] = 1.620, *p* = .205), also indicating no evidence for gender differences regarding perceptual simulation. None of the other interactions involving gender reached significance (all *p*s > .05). Table 1 depicts the mean reaction times for the match and mismatch trials, as well as for the upward and downward trials by age group and gender.

**Table 1:** Means (and standard deviations) of reaction times in match versus mismatch trials and in upward versus downward object movements described in the sentence

Age group	Gender	Mean reaction times (milliseconds)			
		Match (SD)	Mismatch (SD)	Upward movement (SD)	Downward movement (SD)
6-year-olds (*N *= 34)	Male (*N *=17)	1579 (337)	1648 (383)	1580 (351)	1650 (366)
	Female (*N *= 17)	1888 (389)	1874 (390)	1846 (380)	1916 (410)
8-year-olds (*N *=36)	Male (*N *=17)	1145 (214)	1153 (199)	1150 (213)	1147 (211)
	Female (*N *= 19)	1297 (277)	1314 (287)	1302 (289)	1309 (275)
10-year-olds (*N *=37)	Male (*N *=19)	976 (186)	989 (181)	959 (186)	1006 (182)
	Female (*N *= 18)	1055 (198)	1094 (206)	1070 (208)	1075 (192)
Adults (*N *=38)	Male (*N *=7)	669 (70)	737 (59)	706 (61)	700 (59)
	Female (*N *=31)	719 (73)	751 (63)	733 (69)	738 (69)

## Discussion

To investigate whether children’s mental representations during sentence comprehension are dynamic, we developed an experimental design based on Stanfield and Zwaan’s (2001) perceptual mismatch paradigm. This involved a picture verification task that was based on reaction time and, therefore, was independent of the participants’ verbal abilities. The inclusion of both children and adults should contribute to a better understanding of the developmental course of embodied language comprehension.

The initial evidence from earlier adult studies has indicated perceptual simulation of static text information and dynamic text information, such as in the simulation of balls moving toward or away from a protagonist and becoming correspondingly smaller or larger in the simulated perception (Zwaan et al. 2004). The present results for the adult sample support these earlier findings: Adults perceptually simulated the directions of objects’ motions on the vertical axis, confirming earlier findings (described in the introduction) that adults’ mental representations routinely involve dynamic visual aspects (Bergen et al. 2007; Kaschak et al. 2005; Meteyard et al. 2007; Richardson et al. 2003).

Until now, no such statement could be made with respect to children. Previous findings have indicated that children’s mental representations are embodied; for example, they are known to simulate static object information perceptually (Engelen et al. 2011). The present study indicates that children’s mental representations are not only static but also dynamically embodied. In line with Engelen et al. (2011), we found that perceptual simulations also may play a role in sentence comprehension among children. In accordance with these authors, we found no developmental course. Although simulations are elaborate and take time (Barsalou et al. 2008; Louwerse 2013), even the youngest children might have activated motor resonance for comprehension and might have used the representation of dynamic information as a “strategy” for text comprehension. This leads to the assumption that perceptual simulation is developed in the early stages and is also fundamental to children’s comprehension. In addition, our results indicate that children’s perceptual simulation of movement is not limited to protagonist movements (e.g., Author 1999; Barnes et al. 2014; Fecica and O’Neill 2010) but also includes object movements. This generalization is not trivial because it is well known that protagonists play a special role in children’s language comprehension. To give an example, Rall and Harris (2000) demonstrated that children at the age of 3 recall motion verbs (e.g., come vs. go) from the protagonist’s perspective. Furthermore, protagonists’ motivational (Fecica and O’Neill 2010) and emotional (Author 2015) states are incorporated into the situation models of preliterate children. We tentatively conclude that perceptual simulation as a feature of children’s text comprehension is not limited to taking the protagonist’s perspective or might even be independent of that.

Unexpectedly, in our data, reaction times were associated with motion direction. Picture verification was faster when an upward rather than a downward object movement was described in the sentence. A possible explanation for this result would be that upward movements, as well as objects located in the upper perceptual field, tend to be associated with positive emotions, while downward movements and objects located in the lower perceptual field are associated with negative emotions (Casasanto 2009; Casasanto and Dijkstra 2010; Marmolejo-Ramos et al. 2013; Marmolejo-Ramos et al. 2017). Evidence from psycholinguistic research indicates that the emotional valence of word stimuli is related to reaction time in word naming and lexical decision tasks, with positively connoted words being processed faster than negatively connoted ones (referred to as *positivity bias*, e.g., Estes and Adelman 2008; Kuperman et al. 2014; Rodríguez-Ferreiro and Davies 2019). It is possible that this positivity bias also holds for similar tasks at the sentence level. Interestingly, there was a significant difference between age groups with respect to this effect, which was clearly pronounced at age 6 but not in older age groups. This is consistent with recent research, indicating that the positivity bias in word-related tasks is mostly present in preschoolers and decreases with age (Bahn et al. 2017; for a review, see Kauschke et al. 2019). Please note, however, that there was no interaction between motion direction and compatibility. Hence, our study does not provide evidence that the perceptual simulation of vertical movement is influenced by its direction or by the emotional valence that may be associated with it. In addition, the reaction times in the picture verification task yielded a gender difference: Male participants seem to be faster than female ones. Although we do not know for sure, this may reflect gender differences that have been found repeatedly in the field of visuospatial abilities (e.g., Gur et al. 2012). Again, there is no evidence for gender differences with respect to perceptual simulation because gender and compatibility did not interact.

### Limitations and implications for future research

Although the present study identified the perceptual simulation of object motion, this finding must be treated with caution for several reasons. First, although reaction time-based tasks routinely are applied in studies of children, a large variability in variance existed among younger children. This is partly because of their longer reaction times, but it also may indicate that other paradigms should be tested to investigate the development of mental simulation in children. One possible approach would be to access neuropsychology techniques, notably fMRI, as used, for example, by Rueschemeyer et al. (2010), who demonstrated increased blood oxygen level-dependent activity in brain areas associated with motion perception when comprehending motion-sensitive sentences. Furthermore, our results merely indicate that perceptual simulation of vertical motion takes place in a heterogeneous age group ranging from 6 years to adulthood. Although our results do not allow us to determine any development within this broad age range, we cannot say for sure that a specific age group (e.g., 6-year-old children) would perceptually simulate. We encourage further research to replicate our findings with larger samples of younger children, including age 5 and younger to specify the development of perceptual simulation in early childhood (see, for example, Fecica and O’Neill 2010).

It should also be acknowledged that paradigms such as the present approach do not rule out the possibility that the differences in reaction times are based on reconstructive processes that are triggered once motions are seen after the linguistic material. More precisely, someone who sees the picture of a rising or falling object may try to remember whether this object was rising or falling according to the sentence, even if remembering the movement direction was not required. In their research with adults, Stanfield and Zwaan (2001) addressed this problem by replicating their results with an object naming task, which is processed independently from the previously heard sentence. At this point, we state that our research provides a first piece of evidence for dynamic perceptual simulation in children using the picture verification method. Future research may try to replicate these findings with a picture naming task in a children sample.

Moreover, the current study’s design leaves alternative explanations—other than mental simulation—for the differences found between the matching and mismatching conditions. Specifically, a propositional explanation might also be possible. Hearing the sentence “The treasure chest sinks to the seabed,” the participants could have translated it into a propositional form. When they then saw a picture of a treasure chest moving downward, the participants could also have translated this information into a propositional form. Because these two propositional codes match, this could result in shorter reaction times in the match conditions. However, we think that this is rather doubtful. Propositions are abstract representations that have an arbitrary connection to the referent “in the real world” and are, by definition, not analog (Hemforth and Konieczny 2008). Studies have shown that the brain regions that are responsible for processing real motions are also activated during the comprehension of motion-sensitive sentences (e.g., Rueschemeyer et al. 2010). These simulation processes are very precise in the sense that the neurons used for specific motion directions—as opposed to only the mechanisms responsible for processing motion perceptions in general—are activated (cf. Kaschak et al. 2005). These results support the contention that the language comprehension of motion aspects is grounded in perception and action and hence is not merely a construction of proposition-based representations. Furthermore, the recoding of perceptual information in an amodal, proposition-based representation would be quite inefficient for the cognitive system. It is also questionable whether any region of the brain recodes perceptual information into abstract representations (e.g., Barsalou 1999). Therefore, we think that the differences between the matching and mismatching conditions could best be explained by a modal, perceptual-like kind of text comprehension.

We must admit, however, that our study was not designed to directly assess the necessity of perceptual simulation for text comprehension. More than a decade ago, Graesser and colleagues (e.g., de Vega et al. 2008; Graesser and Jackson, 2008) called into question the claim that perceptual simulation would be a prerequisite for coherent text representation. Other researchers have tended to postulate that sensory motor activations indeed are generated initially during language comprehension but are not integrated into the final holistic representation, which is more or less amodal (Hirschfeld et al. 2011). More recent articles have suggested an integrative approach that does not strictly separate modal and amodal representations but that rather assumes that both play a role in language comprehension (e.g., Chatterjee 2010; Dove 2009, 2015; Louwerse and Jeuniaux 2008; Mahon and Caramazza 2008; Willems and Francken 2012; Zwaan 2014). The matter of how these processes may converge should be a key question for future research, especially with regard to children. We recommend the use of external text comprehension measures to explore whether mental simulations enhance explicit and implicit text comprehension and deepen understanding.

Finally, the range of object motions should also be broadened to include the horizontal axis in addition to the vertical axis. Other characteristics of objects moving in space—such as their speed, size, and gravity—also should be included. Because we do not know any previous investigations of object movement simulation in children, we decided to begin from the basic level of sentence processing to explore whether this takes place at all and whether the picture verification task works. In a further step, the simulation of object movements could, for example, be investigated at the text level so that a more natural and ecologically valid scenario of text comprehension would be provided.

## Conclusion

The current study supports previous findings that adults’ situation models routinely involve dynamic visual representations. Language comprehension seems to be grounded in perception and action in adults. We cautiously can extend this finding to children aged between 6 and 10. No indices exist for a developmental time course in children. We assume that grounded cognition is an instrumental process for deep language comprehension, including in children, and may not be a form of cognitive ornamentation.

## Electronic supplementary material

Below is the link to the electronic supplementary material.

Supplementary material 1 (DOCX 23 kb)
