# Correction to: The development of dynamic perceptual simulations during sentence comprehension

**DOI:** 10.1007/s10339-021-01027-4

**Published:** 2021-08-10

**Authors:** Juliane E. K. Hauf, Gerhild Nieding, Benedikt T. Seger

**Affiliations:** Department of Psychology (Developmental Psychology), University of Würzburg, Röntgenring 10, 97070 Würzburg, Germany

## Correction to: Cognitive Processing (2020) 21:197–208 10.1007/s10339-020-00959-7

The article: The development of dynamic perceptual simulations during sentence Comprehension written by Juliane E. K. Hauf, Gerhild Nieding, Benedikt T. Seger, was originally published electronically on the publisher’s internet portal (currently SpringerLink) on 21 February 2021 without open access.

With the author(s)’ decision to opt for Open Choice, the copyright of the article changed on 15 February 2021 © The Author(s) 2021 and the article is forthwith distributed under the terms of the Creative Commons Attribution 4.0 International License (http://creativecommons.org/licenses/by/4.0/), which permits use, duplication, adaptation, distribution and reproduction in any medium or format, as long as you give appropriate credit to the original author(s) and the source, provide a link to the Creative Commons license and indicate if changes were made.

Open Access This article is distributed under the terms of the Creative Commons Attribution 4.0 International License (http://creativecommons.org/licenses/by/4.0/), which permits unrestricted use, distribution and reproduction in any medium, provided you give appropriate credit to the original author(s) and the source, provide a link to the Creative Commons license and indicate if changes were made.

The original article was corrected.

