# Supplementary material for: The development of dynamic perceptual simulations during sentence comprehension
Source: Cogn Process. 2020 Feb 21;21(2):197–208. doi: 10.1007/s10339-020-00959-7 (PMC8354901; doi:10.1007/s10339-020-00959-7)
Supplement: Supplementary file 1 — Supplementary material 1 (DOCX 23 kb) [file 10339_2020_959_MOESM1_ESM.docx]

Appendix

*Table A1.* List of experimental sentences used in the study.

| Direction | Matching Condition | German Original | English Translation |
| --- | --- | --- | --- |
| Down | A | Der *Apfel* fällt hinunter auf das Gras. | The *apple* falls on the grass. |
| Down | A | Tamara wirft das *Kissen* hinunter auf den Teppichboden. | Tamara throws the *pillow* on the carpet. |
| Down | A | Der *Stein* fällt hinein ins Wasser. | The *stone* falls into the water. |
| Down | A | Die *Katze* springt hinunter auf die Straße. | The *cat* jumps down on the street. |
| Down | A | Der *Würfel* fällt hinunter auf den Boden. | The *die* falls on the floor. |
| Down | A | Der *Fahrradhelm* fliegt runter auf den Fußboden. | The *bike helmet* drops on the ground. |
| Down | A | Die *Pflaume* fällt hinunter auf die Wiese. | The *plum* falls on the meadow. |
| Down | A | Der *Tannenzapfen* fällt hinunter auf den Waldboden. | The *fir cone* falls on the forest soil. |
| Down | A | Der *Anker* sinkt hinab auf den Meeresboden. | The *anchor* sinks to the seabed. |
| Down | B | Die *Regentropfen* prasseln auf das Dach. | *Rain drops* are drumming on the roof. |
| Down | B | Das *Schiff* sinkt herab im Ozean. | The *ship* sinks into the ocean. |
| Down | B | Das *Buch* fliegt hinunter auf den Boden. | The *book* drops on the floor. |
| Down | B | Die *Schatztruhe* sinkt herab auf den Meeresboden. | The *treasure chest* sinks onto the seabed. |
| Down | B | Der *Wecker* fällt hinunter auf den Fußboden. | The *alarm* falls on the ground. |
| Down | B | Die *Schere* fällt hinunter auf den Boden. | The *scissors* fall on the floor. |
| Down | B | Der *Weihnachtsmann* rutscht durch den Schornstein hinunter. | *Santa* slides down through the chimney. |
| Down | B | Die *Spinne* seilt sich herab. | The *spider* ropes down. |
| Down | B | Der *Regenwurm* fällt aus dem Vogelschnabel. | The *earthworm* falls out of the beak. |
|  |  |  |  |
| *Table A1 (continued).* | | | |
| Direction | Matching Condition | German Original | English Translation |
| Up | A | Der *Hubschrauber* fliegt in die Luft. | The *helicopter* flies into the air. |
| Up | A | Claudia wirft den *Ball* in die Höhe. | Claudia throws the *ball* up in the air. |
| Up | A | Der *Luftballon* fliegt hoch in die Luft. | The *balloon* flies up into the air. |
| Up | A | Die *Rakete* fliegt hoch ins Weltall. | The *rocket* starts into space. |
| Up | A | Lisa stellt die *Tasse* hoch auf das Regal. | Lisa puts the *cup* on the cupboard. |
| Up | A | Tim wirft den *Teddybären* hinauf auf das Hochbett. | Tim throws the *teddy bear* on the loft bed. |
| Up | A | Vanessa wirft die *Puppe* hoch. | Vanessa throws up the *doll*. |
| Up | A | Der *Heißluftballon* steigt hoch in die Wolken. | The *hot-air balloon* rises up into the clouds. |
| Up | A | Die *Feuerwerksrakete* fliegt hoch in den Himmel. | The *firework rocket* streaks heavenwards. |
| Up | B | Der *Drachen* steigt hinauf in den Himmel. | The *kite* rises to the sky. |
| Up | B | Der Kran zieht das *Auto* nach oben aus dem Wasser. | The crane pulls the *car* out of the water. |
| Up | B | Lukas zieht die *Toastscheibe* aus dem Toaster. | Luke pulls the *toast slice* out of the toaster. |
| Up | B | Der Lehrer schiebt die *Tafel* nach oben. | The teacher pushes up the *blackboard*. |
| Up | B | Katrin hängt die *Kugel* hoch an den Weihnachtsbaum. | Catherine hangs the *glitter ball* on the Christmas tree. |
| Up | B | Martin hebt die *Muschel* auf. | Martin picks up the *shell*. |
| Up | B | Der Angler zieht einen *Fisch* aus dem Wasser. | The angler pulls a *fish* out of the water. |
| Up | B | Die Braut wirft den *Blumenstrauß* in die Höhe. | The bride throws up the *bouquet*. |
| Up | B | Der Schüler streckt die *Hand* hoch. | The student holds up his *hand*. |

Emphasized words refer to the target object in the picture verification task.

*Table A2.* Practice and filler sentences used in the study.

| Function | Task Type | German Original | English Translation |
| --- | --- | --- | --- |
| Practice | No/Down | Die Schaufel liegt neben dem Sandkasten. | The shovel lies next to the sand box. |
| Practice | No/Down | Das Telefon liegt neben dem Telefonbuch. | The telephone lies next to the phone book. |
| Practice | Yes/Down | Der *Tee* ist in der Tasse. | There is *tea* in the teacup. |
| Practice | No/Up | Die Luftmatratze schwimmt auf dem Wasser. | The air mattress floats on the water. |
| Practice | No/Up | Die Balletttänzerin tanzt auf der Bühne. | The danseuse dances on stage. |
| Practice | Yes/Up | Der *Kindersitz* ist hinten im Auto. | The *children’s seat* is at the back of the car. |
| Practice | Yes/Up | Der *Frosch* hüpft hinunter auf die Seerose. | The *frog* leaps down on the water lily. |
| Practice | Yes/Down | Der *Floh* hüpft auf das Bett hinauf. | The *flea* hops up onto the bed. |
| Filler | Yes/Down | Maike riecht an der *Blume*. | Maike smells at the *flower*. |
| Filler | Yes/Down | Maria sieht einen *Hund*. | Mary sees a *dog*. |
| Filler | Yes/Down | Der *Ball* rollt langsam ins Tor. | The *football* rolls slowly to the goal. |
| Filler | Yes/Down | *Tom* wirft einen Schneeball direkt ans Fenster. | *Tom* throws a snowball straightly to the window. |
| Filler | Yes/Down | Der *Kreisel* dreht sich schnell auf dem Tisch. | The *spin top* spins fast on the table. |
| Filler | Yes/Up | Laura mischt sorgfältig das *Kartenspiel*. | Laura attentively shuffles the *cards*. |
| Filler | Yes/Up | Dominik flitzt schnell zum *Schulbus*. | Dominik dashes to the *school bus*. |
| Filler | Yes/Up | Das Kissen liegt auf dem *Bett*. | The pillow lies on the *bed*. |
| Filler | Yes/Up | Das Handy ist in der *Handtasche*. | The cell phone is in the *handbag*. |
| Filler | Yes/Up | Das Wasser ist im *Eimer*. | There is water in the *bucket*. |
| Filler | No/Down | Das Kaninchen hoppelt quer über die Wiese. | The rabbit scuttles across the meadow. |
| Filler | No/Down | Das Ferkel suhlt sich vergnügt im Schlamm. | The piglet cheerfully wallows in the mud. |
| Filler | No/Down | Der Hund tollt voller Freude auf der Wiese herum. | The dog joyfully frolics around the meadow. |
| Filler | No/Down | Der Grashüpfer springt von Grashalm zu Grashalm. | The grasshopper jumps from culm to culm. |
| *Table A2 (continued).* | | | |
| Function | Task Type | German Original | English Translation |
| Filler | No/Down | Der Hund schnuppert hungrig an seinem Futter. | The dog sniffs hungrily at its food. |
| Filler | No/Down | Die Katze jagt einer Maus hinterher. | The cat chases after a mouse. |
| Filler | No/Down | Das Pferd springt weit über das Hindernis. | The horse greatly leaps the hurdle. |
| Filler | No/Down | Der Ball rollt quer über die Straße. | The ball rolls across the road. |
| Filler | No/Down | Das Ei rollt aus dem Nest. | The egg rolls out of its nest. |
| Filler | No/Down | Der Hase hüpft ins Gras hinein. | The hare leaps into the grass. |
| Filler | No/Down | Die Bowlingkugel rollt über die Bahn. | The bowling ball rolls across the alley. |
| Filler | No/Down | Der Wurm kriecht langsam auf die Straße. | The worm creeps slowly on the road. |
| Filler | No/Down | Die Ente paddelt auf dem Teich entlang. | The duck paddles along the pond. |
| Filler | No/Down | Der Fisch schwimmt im Wasser herum. | The fish swims around in the water. |
| Filler | No/Down | Die Fernbedienung liegt neben dem Fernseher. | The zapper lies next to the TV. |
| Filler | No/Down | Das Baby krabbelt auf der Decke herum. | The baby crawls on the blanket. |
| Filler | No/Down | Der Schlittschuhläufer gleitet über das Eis. | The skater glides over the ice. |
| Filler | No/Down | Die Mutter schiebt das Blech hinein in den Ofen. | Mother pushes the griddle into the oven. |
| Filler | No/Down | Der Krebs krabbelt über den Sand. | The crab crawls across the sand. |
| Filler | No/Down | Das Boot segelt über das Wasser. | The boat sails across the water. |
| Filler | No/Down | Der Hund rennt schnell über die Wiese. | The dog runs quickly across the meadow. |
| Filler | No/Down | Das Kind schüttelt die Rassel. | The child shakes the rattle. |
| Filler | No/Down | Der Clown tanzt fröhlich in der Manege. | The clown happily dances in the circus ring. |
| Filler | No/Up | Der Vogel sitzt quietschvergnügt auf der Fensterbank. | Bouncily the bird sits on the window sill. |
| Filler | No/Up | Die Banane liegt in der Obstschale. | The banana lies in the fruit bowl. |
| Filler | No/Up | Die Erdbeere ist im Kühlschrank. | The strawberry is in the fridge. |
| *Table A2 (continued).* | | | |
| Function | Task Type | German Original | English Translation |
| Filler | No/Up | Der Stift ist im Stiftebecher. | The pen is in the pencil cup. |
| Filler | No/Up | Die Spaghetti sind auf dem Teller. | The spaghetti are in the plate. |
| Filler | No/Up | Der Schlüssel hängt am Schlüsselhaken. | The key hangs on the key rack. |
| Filler | No/Up | Der Igel sitzt unter dem Laubhaufen. | The hog sits under a pile of leaves. |
| Filler | No/Up | Der Hahn sitzt auf dem Heuhaufen. | The cock sits on the haystack. |
| Filler | No/Up | Die Leiter steht im Keller. | The ladder stands in the basement. |
| Filler | No/Up | Der Staubsauger steht im Putzschrank. | The hoover stands in the closet. |
| Filler | No/Up | Der Taschenrechner ist in der Schultasche. | The calculator is inside the school bag. |
| Filler | No/Up | Die Zwiebel ist im Gemüsekorb. | The onion is in the vegetable basket. |
| Filler | No/Up | Das Spiegelei ist in der Pfanne. | The fried egg is in the pan. |
| Filler | No/Up | Die Tomate ist auf der Pizza. | The tomato is on the pizza. |
| Filler | No/Up | Die Schuhe stehen vor der Tür. | The shoes stand outside the door. |
| Filler | No/Up | Das Dreirad steht in der Garage. | The tricycle stands in the garage. |
| Filler | No/Up | Der Biber springt über den Baumstamm hinüber. | The beaver jumps over the log. |
| Filler | No/Up | Der Marienkäfer sitzt ruhig auf einem Blatt. | The ladybird calmly sits on a leaf. |
| Filler | No/Up | Der Löwe läuft im Käfig auf und ab. | The lion walks up and down the cage. |
| Filler | No/Up | Das Pony springt über den Kasten. | The pony leaps over the vaulting box. |
| Filler | No/Up | Die Zahnbürste liegt auf dem Waschbecken. | The toothbrush lies on the sink. |
| Filler | No/Up | Der Kugelschreiber liegt im Mäppchen. | The ball pen lies in the pencil case. |
| Filler | No/Up | Die Gießkanne steht neben dem Gartenhäuschen. | The watering can stands next to the garden shed. |

Task type indicates whether the object in the picture verification task was mentioned in the sentence (“Yes”) or not (“No”) and whether the picture animation moves up or down. For “yes” trials, words referring to target objects are emphasized.
